# Targeting NRF2 uncovered an intrinsic susceptibility of acute myeloid leukemia cells to ferroptosis

**DOI:** 10.1186/s40164-023-00411-4

**Published:** 2023-05-17

**Authors:** Xin Liu, Shuxin Zhong, Kangjie Qiu, Xi Chen, Weiyue Wu, Jiamian Zheng, Yanwen Liu, Haolong Wu, Shiyun Fan, Dingrui Nie, Xianfeng Wang, Zhi Yu, Ziwei Liao, Mengjun Zhong, Yangqiu Li, Chengwu Zeng

**Affiliations:** 1 Key Laboratory for Regenerative Medicine of Ministry of Education, Institute of Hematology, School of medicine, Jinan University, No.601, West Huangpu Avenue, Guangzhou, 510632 P.R. China; 2 Department of Medical Biochemistry and Molecular Biology, School of Medicine, Jinan University, Guangzhou, P.R. China; 3 Department of Hematology, First Affiliated Hospital, Jinan University, Guangzhou, 510632 P.R. China; 4 Department of Hematology, Guangzhou Women and Children’s Medical Center, Guangzhou, 510623 P.R. China

**Keywords:** NRF2, GPX4, Acute myeloid leukemia, Ferroptosis

## Abstract

Drug resistance and poor treatment response are major obstacles to the effective treatment of acute myeloid leukemia (AML). A deeper understanding of the mechanisms regulating drug resistance and response genes in AML is therefore urgently needed. Our previous research has highlighted the important role of nuclear factor E2-related factor 2 (NRF2) in AML, where it plays a critical role in detoxifying reactive oxygen species and influencing sensitivity to chemotherapy. In this study, we identify a core set of direct NRF2 targets that are involved in ferroptosis, a novel form of cell death. Of particular interest, we find that glutathione peroxidase 4 (GPX4) is a key ferroptosis gene that is consistently upregulated in AML, and high expression of GPX4 is associated with poor prognosis for AML patients. Importantly, simultaneous inhibition of NRF2 with ML385 and GPX4 with FIN56 or RSL3 synergistically targets AML cells, triggering ferroptosis. Treatment with ML385 + FIN56/RSL3 resulted in a marked reduction in NRF2 and GPX4 expression. Furthermore, NRF2 knockdown enhanced the sensitivity of AML cells to the ferroptosis inducers. Taken together, our results suggest that combination therapy targeting both NRF2 and GPX4 may represent a promising approach for the treatment of AML.

**Supplementary Information:**

The online version contains supplementary material available at 10.1186/s40164-023-00411-4.

## Introduction

Acute myeloid leukemia (AML) is the most common type of leukemia in adults, characterized by the uncontrolled proliferation of myeloid cells in the blood, bone marrow, and other tissues [1–4]. Despite recent advancements in treatment, AML patients with recurrent genetic abnormalities still have a poor prognosis, and relapsed or refractory AML poses a significant challenge[5]. Fortunately, in recent years, new drugs have emerged that provide promising options for relapsed or refractory AML treatment, and the search for novel targeted therapies offers hope for effective combination strategies against AML [6, 7].

Nuclear factor E2-related factor 2 (NRF2) is a crucial regulator in the anti-oxidative stress pathway [8]. However, recent studies have shown that enhanced reactive oxygen species (ROS) detoxification and other NRF2 functions can be pro-tumorigenic [9, 10]. Activating mutations in NRF2 and KEAP1 can promote tumor growth in several types of cancer [11, 12]. It was recently proposed that the NRF2-mediated pathway is a common survival pathway in AML [13]. In addition, studies have suggested that increased NRF2 expression mediated by oncogenes is a novel mechanism for activating the NRF2 antioxidant program in solid tumors [9]. Although many genes are known to be regulated by NRF2 in cancer cells, the direct targets of NRF2 are not yet fully understood [14, 15]. Therefore, a better understanding of the NRF2 pathway could pave the way for new therapeutic interventions in cancer treatment.

Ferroptosis is a newly identified form of non-apoptotic cell death that is characterized by iron-dependent activation of lipoxygenase and subsequent lipid peroxidation (LPO), and it has emerged as a critical factor in various types of cancers [16–20], including leukemia [21]. Ferroptosis-induced cancer cell death presents a promising opportunity for anticancer therapy [14, 22]. One key regulator of cell survival and growth is NRF2, which induces multiple antioxidant genes, including glutathione peroxidase 4 (GPX4) [14]. GPX4 is a central defense enzyme that plays a critical role in catalyzing lethal lipid peroxide into nontoxic lipid alcohol to counteract LPO and ferroptosis [23, 24]. Interestingly, previous studies have highlighted the critical role of NRF2 in ROS detoxification and chemotherapy sensitivity in AML. However, the precise mechanisms underlying the regulation of ferroptosis by NRF2 in AML remained poorly understood until this study. In addition, the regulatory and functional roles of GPX4 in the treatment response of AML cells have not been fully explored.

In this study, we identified a novel mechanism by which NRF2 activation leads to overexpression of GPX4 in AML, resulting in chemoresistance and evasion of ferroptosis. By using various ferroptosis inducers and inhibitors, they have shown that NRF2-deficient AML cells are particularly sensitive to ferroptosis inducers and that NRF2-induced GPX4 supports AML cell survival against drug treatment. The study also demonstrates that combined inhibition of NRF2 and GPX4 can significantly reduce the expression of both genes, thereby facilitating ferroptosis. These findings provide important insights into the mechanism of chemoresistance in AML and suggest that targeting NRF2 and GPX4 may be a promising therapeutic strategy for the treatment of this disease.

## Materials and methods

### Clinical samples

Bone marrow (BM) samples were obtained from sixteen patients with newly diagnosed AML (characteristics provided in the Supplemental Table 1). Peripheral blood (PB) samples were obtained from eight healthy individuals (HIs). All samples were obtained with written informed consent in accordance with the ethical committee of the First Affiliated Hospitals of Jinan University.

### Cell lines and reagents

AML cell lines HL60, MV4;11, KG1a, U937, HEL, Molm13, NB4, KG1 and Kasumi-1 were cultured in RPMI 1640 (Invitrogen) containing 10% fetal bovine serum (FBS). HEK293T cells were cultured in DMEM (Invitrogen) medium containing 10% FBS. All these cell lines were cultured in a humidified atmosphere containing 5% CO_2_ at 37 °C. Ferrostatin-1, ML385, FIN56, and RSL3 were purchased from Selleck Chemicals (Houston, TX, USA).

### Establishment of stable cell lines

Specific oligonucleotides directed against human NRF2 were designed and cloned into the pLKO.1-puro plasmid according to the protocol recommended by Addgene, and empty pLKO.1-puro plasmid served as control. Lentiviruses carrying pLKO.1-puro were prepared by the transfection of standard packaging vectors into HEK293T cells using lipofectamine 3000 (Life Technologies, USA). Viral supernatant was harvested 24 and 48 h after transfection. A total of 2 × 10^5^ MV4;11 cells were co-cultured with virus supernatant plus 8 µg/mL polybrene (Sigma-Aldrich, St Louis, USA). Then 5 µg/mL puromycin was applied to select positively infected cells for at least 5 days. The shRNA sequences are listed in Supplemental Material 1. For ectopic expression of NRF2, lentiviruses overexpressing NRF2 (LV-NRF2) and empty viruses (LV-NC) were purchased from Genechem (Shanghai, China). A total of 5 µg/mL puromycin was used to select positively infected cells for at least 5 days.

### Detection of cell cycle and cell death

For analysis of cell death, cells were harvested, and the Annexin-V-APC/PI Detection Kit (MultiSciences, Shanghai, China) was used following the manufacturer’s protocols and performed as previously described [25, 26]. For cell cycle assay, the Cell Cycle Staining Kit (MultiSciences, Shanghai, China) was used according to the manufacturer’s instructions. Cells were collected by CytoFLEX, and data were analyzed by Flowjo software.

### Quantitative real-time PCR analysis

Total RNA from cells were extracted using TRIzol reagent (Invitrogen, CA, USA) following the manufacturer’s instructions and was reversed transcribed into cDNA using High-Capacity cDNA Reverse Transcription Kits (Applied Biosystems, CA, USA). Quantitative real-time PCR (qRT-PCR) was performed with SYBR Green (Tiangen, China) in a total volume of 20 µl using the following program: 95°C for 5 min followed by 40 cycles of 95℃ for 30s, 60℃ for 30s, and 72℃ for 30s. Relative target gene expression levels were normalized to the mRNA level of β-actin. The primers for qRT-PCR are shown in Supplemental Material 1.

### Western blotting

Cells were collected and lysed in 1х SDS loading buffer containing protease inhibitors. Protein lysates were separated using 12% SDS-PAGE and subsequently transferred to polyvinylidene fluoride membrane (Millipore, USA) following by blocking with QuickBlock™ blocking buffer (Beyotime, China) and incubation with antibodies directed against GPX4 (1:1000, 52,455, CST, MA, USA) or NRF2 (1:800, sc-365,949, Santa cruz) overnight at 4℃. Anti-β-actin antibody (1:5,000, Beyotime, China) served as control. After incubation with a secondary antibody, ECL (Beyotime, China) was used to amplify the binding antibody signal, and images were obtained with a UVITEC photo documenter. The full gel images in the paper figures are displayed in Figure S1.

### Small interfering RNA (siRNA) transfection

The Neon® Transfection System (Invitrogen) was used to transfect 100 pmol oligonucleotides into MV4;11 cells in a total volume of 10 µl [27, 28]. In brief, 2 × 10^5^ cells were suspended in a volume of 10 µl with 100 pmol oligonucleotides and transfected three times in each 12-well plate. After electroporation, cells were cultured in RPMI 1640 medium supplemented with 10% FBS at 37 °C and 5% CO_2_ for 24 h, and subsequently divided into two groups with or without drug treatment. The cells were then collected for protein level detection and evaluation of cell death. Supplemental Material 1 lists the siRNA sequences targeting GPX4.

### Measurement of lipid peroxidation (LPO)

To detect LPO, C11-BODIPY 581/591 (Abclonal, China) was employed following the manufacturer’s instructions. In brief, cells were labeled with C11-BODIPY (2.5 µM) in 400 µL RPMI 1640 medium and incubated at 37 °C and 5% CO_2_ in a cell incubator away from light for 60 min. The cells were then washed twice with PBS, resuspended in 200 µL of fresh PBS, and analyzed using flow cytometry.

### RNA-seq analysis

RNA from LV-NC and LV-NRF2 MV4;11 cells was extracted using TRIzol reagent. Amplified cDNA was sequentially converted into short-read sequencing libraries using the VAHTS Universal V6 RNA-seq Library Prep Kit for Illumina® Hisat2 (version: 2.0.4). Sequencing was performed by Novogene Co. Ltd., Tianjin Biotechnology Corporation. The expression level of each gene was calculated as fragments per kilobase of exon model per million mapped reads (FPKM).

### Statistical analysis

Unless otherwise stated in the figure legends, all data are presented as the mean ± SD of three independent experiments. Statistical analysis was conducted using SPSS 22.0 software on data from independent biological replicates. Student’s t-test was used to determine the significance of differences between two groups. One-way ANOVA followed by either Bonferroni or Dunnett’s post hoc test was applied to compare three or more groups, as specified in the figure legends. A *p* value < 0.05 was considered significant. *, *p* < 0.05, **, *p* < 0.01, ***, *p* < 0.001.

## Results

### NRF2 target gene analysis reveals its role in ferroptosis

**Fig. 1:**
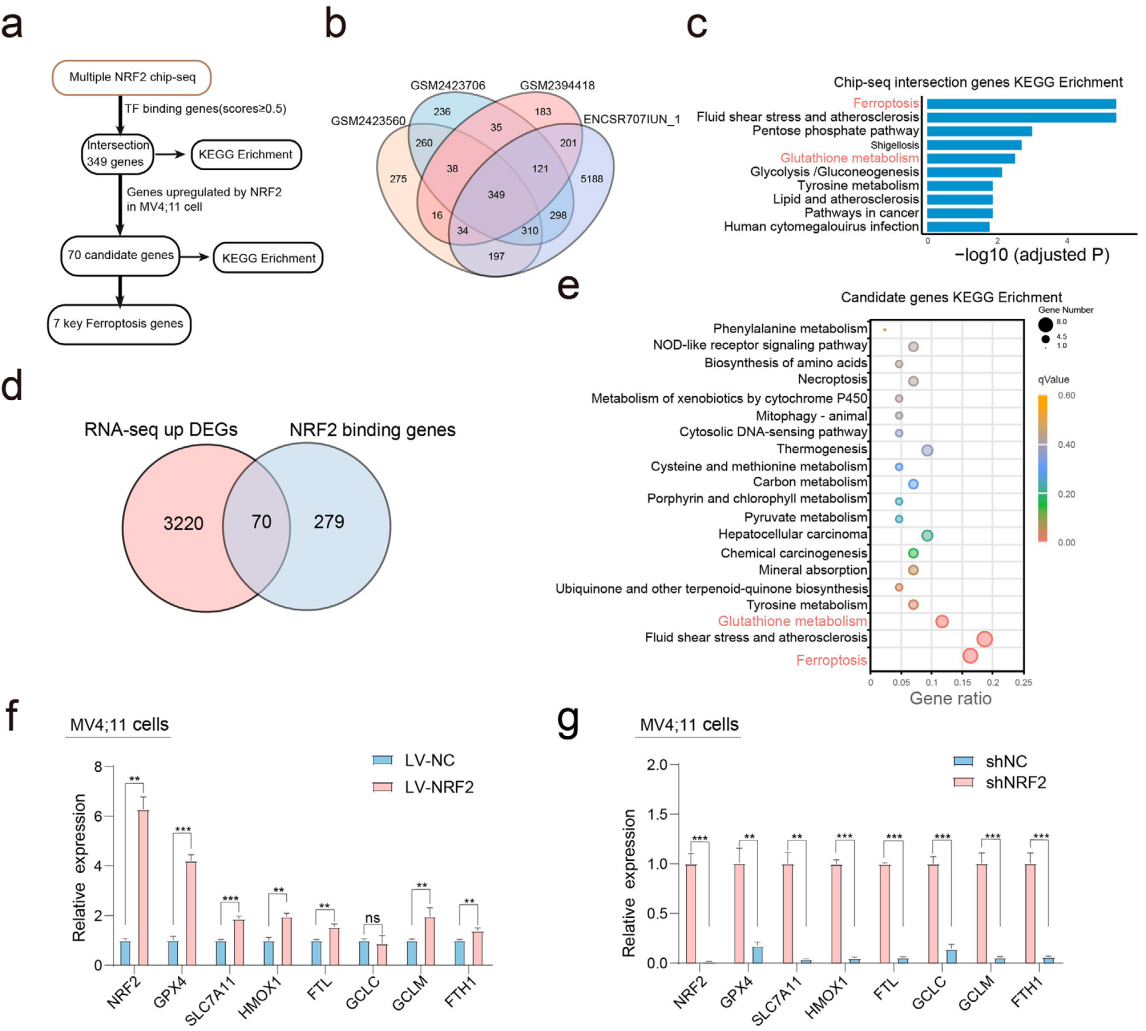
Antioxidant and ferroptosis genes are validated as direct NRF2 targets. (**a**) The flow chart for screening potential NRF2 target genes is depicted, indicating the various steps involved in identifying and validating NRF2 target genes. (**b**) A Venn diagram analysis of four NRF2 Chip-seq cohorts is shown, indicating the overlap and unique genes identified by each cohort. (**c**) The top 10 enriched terms of the Kyoto Encyclopedia of Genes and Genomes (KEGG) pathway analyses of Chip-seq intersection genes are displayed, highlighting the most significant biological processes and pathways associated with NRF2 target genes. (**d**) A Venn diagram shows the overlapping target genes between NRF2 Chip-seq target genes and RNA-seq up-regulated genes, indicating potential candidate genes for further analysis. (**e**) A scatter plot of the top 20 enriched KEGG pathways of LV-NRF2 vs. LV-NC is shown in the bubble plot, highlighting the pathways significantly enriched in NRF2-overexpressing cells. (**f**) The relative expression of genes related to ferroptosis in NRF2-overexpressing MV4;11 cells were detected by qRT-PCR. (**g**) The expression of genes related to ferroptosis in NRF2-knockdown MV4;11 cells were detected by qRT-PCR. Data are expressed as the mean ± SD. *n* = 3 or more independent biological replicates, presented as individual points. P value < 0.05 was considered significant (f-g, two-tailed unpaired Student’s t test).

Our recent studies have demonstrated that NRF2 promotes the expression of key molecules involved in AML development and drug sensitivity. To identify potential therapeutic targets for AML, we performed a broad screening of direct NRF2 target genes (Fig. 1a). Our hypothesis was that NRF2 target genes would contain ChIP-Seq binding peaks in their vicinity and show altered expression levels in RNA-seq studies. To generate a list of NRF2 target genes, we initially utilized several ChIP-Seq profiling datasets (ENCSR707IUN_1, GSM2394418, GSM2423706, GSM2423560) and identified 349 unique overlapping genes. These represent a high-confidence set of NRF2-bound genomic regions (Fig. 1b). The majority of the identified binding sites were found near potential NRF2 target genes, suggesting that these genes could be regulated by NRF2. We further performed KEGG pathway analysis on these potential NRF2 target genes and found that they were significantly enriched in several terms, including ferroptosis and glutathione metabolism, both of which are closely related to ferroptosis (Fig. 1c, Supplemental Material 2). Next, we conducted RNA-seq to identify NRF2 target genes in AML. We identified over 3,000 upregulated genes in NRF2-overexpressing MV4;11 cells (log2 fold change ≥ 0.25). Our results revealed the upregulation of numerous antioxidative and ferroptosis genes in response to NRF2 overexpression. Subsequently, we identified 70 unique genes overlapping between ChIP-Seq and RNA-seq (Fig. 1d), and compiled them in Supplemental Material 3. Consistent with the previous findings, we found that the NRF2 targets are mainly involved in the processes of ferroptosis and glutathione metabolism (Fig. 1e, Supplemental Material 4). Both NRF2 and ferroptosis have been shown to be linked to cancer therapy resistance. It is intriguing to speculate that these NRF2 targets may act in concert to function as anti-ferroptosis mediators upon drug treatment in AML.

Using qRT-PCR analysis, we validated that several of these genes are indeed transcriptional targets of NRF2 in AML cells (Fig. 1f-g). Among the selected genes, including SLC7A11, GCLC, GPX4, FTH1, HMOX1, GCLM, and FTL, GPX4 mRNA was upregulated upon lentivirus-mediated NRF2 overexpression and downregulated upon shRNA-mediated NRF2 (shNRF2) knockdown. Furthermore, GPX4 was also upregulated in response to NRF2 activation by arsenic trioxide (ATO) and cytarabine (Ara-C) treatment (Figure S2). Given the known activation of NRF2 in AML and its resistance to therapies, we propose that GPX4 may be one of the key targets that confer an anti-ferroptosis role to NRF2.

### NRF2-regulated GPX4 was increased and associated with poor prognosis in AML

**Fig. 2:**
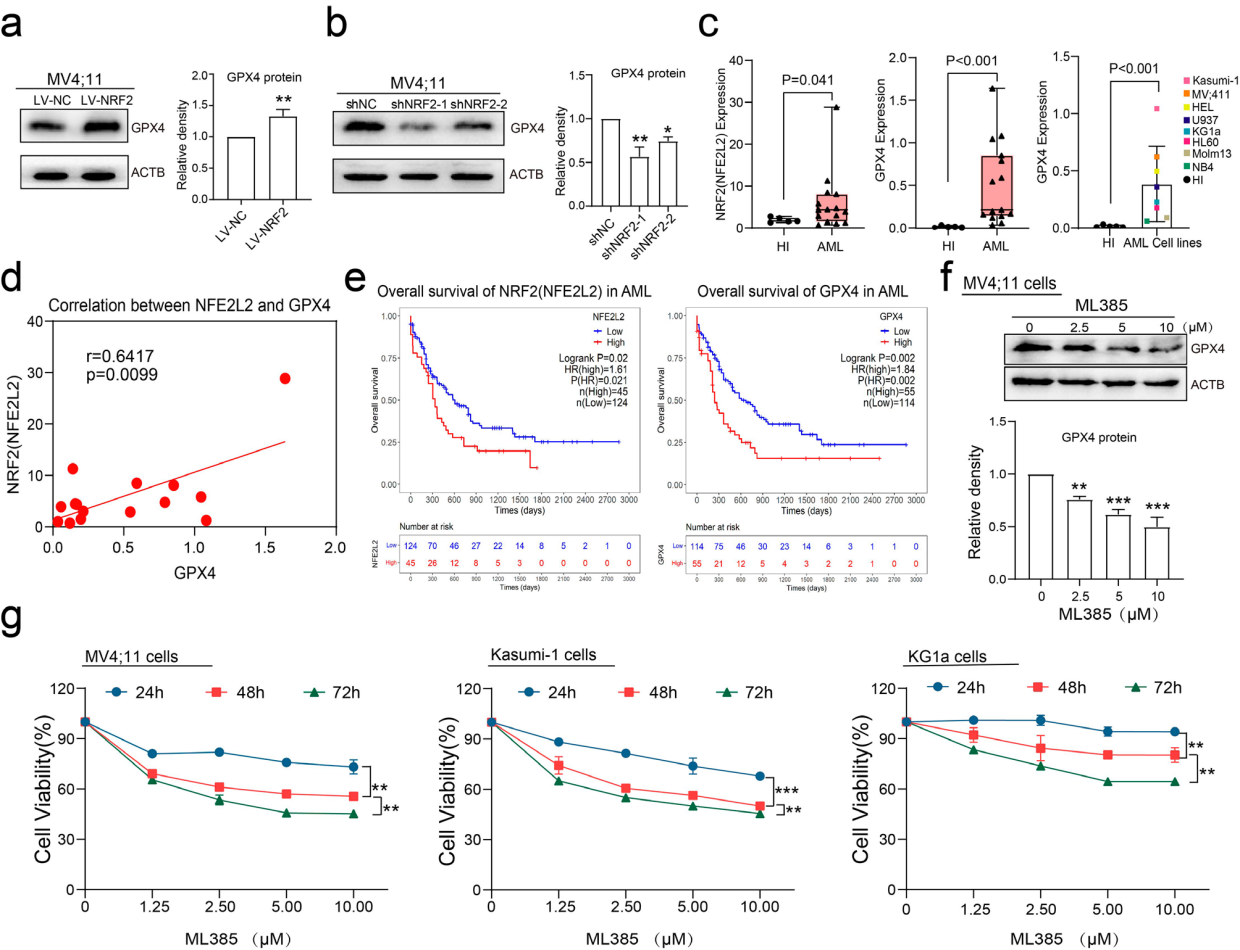
NRF2-regulated GPX4 was increased and associated with poor prognosis in AML. (**a**) The expression of GPX4 in MV4;11 cells transfected with LV-NC or LV-NRF2 was detected by western blotting. (**b**) The expression of GPX4 in MV4;11 cells transfected with shNC or shNRF2 was detected by western blotting. (**c**) The expression levels of NRF2(NFE2L2) (left panel) and GPX4 (middle panel) in HIs’ PBMCs and AML patients’ bone marrow mononuclear cells (BMMCs) were detected by qRT-PCR, and the GPX4 expression in AML cell lines (right panel) was detected by qRT-PCR. (**d**) Spearman correlation analysis of the NFE2L2 and GPX4 expression in AML patients’ BMMCs. (**e**) Overall survival analysis was performed on high-NFE2L2 or low-NFE2L2 and high-GPX4 or low-GPX4 expression groups in the AML patient cohorts in TCGA dataset, indicating the potential prognostic value of NRF2 and GPX4 expression in AML patients. (**f**) MV4;11 cells were treated with the indicated concentrations of ML385 for 24 h, and the expression of GPX4 was detected by western blotting. (**g**) MV4;11, Kasumi-1, and KG1α cells were administered with the indicated concentrations of ML385 for 24, 48, 72 h, and cell viability was detected by the CCK8 assay. Data are expressed as mean ± SD. *n* = 3 or more independent biological replicates, presented as individual points. P value < 0.05 was considered significant (a, c, two-tailed unpaired Student’s t test; b, f, one-way ANOVA with Dunnett’s post hoc test; e, log-rank test; g, one-way ANOVA with Bonferroni post hoc test).

To confirm the regulatory role of NRF2 on GPX4 in AML, we used western blotting to detect changes in GPX4 expression after manipulating NRF2 expression. As shown in Fig. 2a and b, overexpression of NRF2 led to increased GPX4 expression, while knockdown of NRF2 resulted in decreased GPX4 expression. Moreover, we observed higher expression of NRF2 and GPX4 in the BM of AML patients compared with HIs (Fig. 2c). Among several AML cell lines, we found high expression of GPX4 in MV4;11 and Kasumi-1 cells (Fig. 2c, right panel), which were subsequently used in our experiments. We also analyzed the expression of NRF2 and GPX4 in 15 AML patients and found a positive correlation between the two (Fig. 2d). Furthermore, survival analysis of AML patients included in The Cancer Genome Atlas (TCGA) dataset showed that both NRF2 and GPX4 expression were significantly associated with poor overall survival (OS) (Fig. 2e and Figure S3a-b), indicating that their increased expression is associated with poor prognosis in AML. In addition, the NRF2 inhibitor ML385 reduced GPX4 protein levels in a concentration-dependent manner (Fig. 2f). We also found that inhibiting NRF2 with ML385 inhibited the viability of various AML cell lines (Fig. 2g), suggesting that NRF2 and GPX4 could represent promising targets for the treatment of AML cancer cells.

### NRF2 inhibition (ML385) cooperates with GPX4 inhibition (FIN56/RSL3) to reduce the viability and induce the cell death of AML cells

**Fig. 3:**
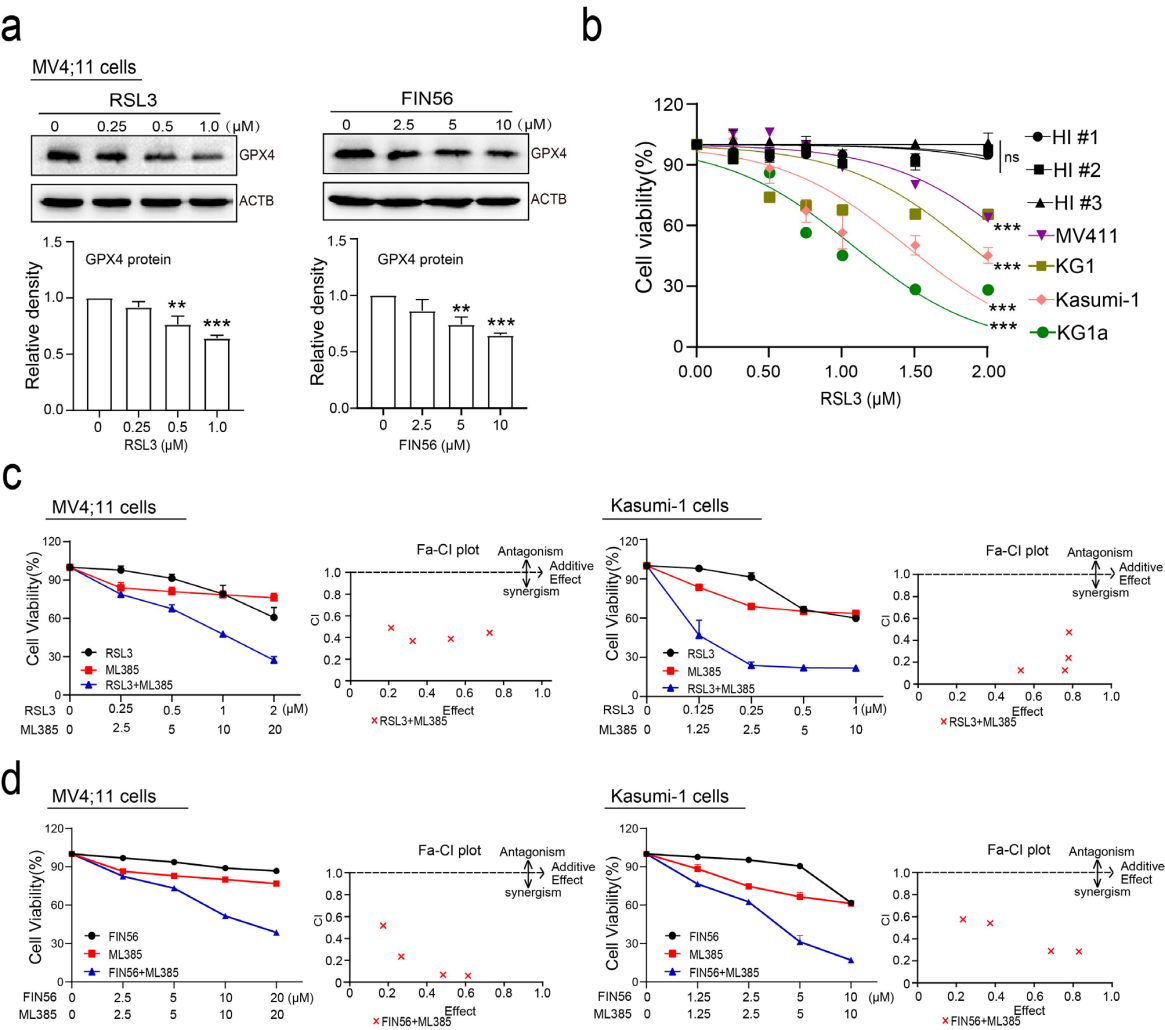
ML385 cooperates with FIN56/RSL3 to reduce the viability of AML cells. (**a**) MV4;11 cells were treated with the indicated concentrations of RSL3 (left panel) or FIN56 (right panel) for 24 h, and the expression of GPX4 was detected by western blotting. (**b**) AML cell lines and PBMCs from HIs were treated with the indicated concentrations of RSL3 for 24 h, and the cell viability was detected by the CCK8 assay. (**c**) MV4;11 (left panel) and Kasumi-1 cells (right panel) were treated with ML385 alone, RSL3 alone, or the combination of ML385 and RSL3 at the indicated concentrations for 24 h, and the cell viability was measured by CCK8 assay. CI values were calculated by the Chou-Talalay method. The dashed line designates a CI value of 1, and CI < 1 indicates a synergistic interaction between the two agents in the combination. (**d**) MV4;11 (left panel) and Kasumi-1 cells (right panel) were treated with ML385 alone, FIN56 alone, or the combination of ML385 and FIN56 at the indicated concentrations for 24 h, and the cell viability was measured by CCK8 assay. CI values were calculated by the Chou-Talalay method. The dashed line designates a CI value of 1, and CI < 1 indicates a synergistic interaction between the two agents in the combination. Data are expressed as the mean ± SD. *n* = 3 or more independent biological replicates, presented as individual points. P value < 0.05 was considered significant (a, one-way ANOVA with Bonferroni post hoc test; b, two-tailed unpaired Student’s t test).

It has been reported that RSL3 and FIN56 can specifically inhibit GPX4 to induce the ferroptosis of tumor cells [14, 29, 30]. To investigate the synergistic effects of NRF2 and GPX4 inhibition in AML cells, we first explored the use of these specific GPX4 inhibitors to induce ferroptosis in AML cells. Our experiments showed that treatment with GPX4 inhibitors resulted in a dose-dependent suppression of GPX4 expression in MV4;11 cells (Fig. 3a). We also found that RSL3 inhibited the viability of various AML cell lines in a concentration-dependent manner, while having little effect on peripheral blood mononuclear cells (PBMCs) from HIs, indicating its relative safety (Fig. 3b). Next, AML cell lines MV4;11, Kasumi-1, and KG1α were treated with NRF2 inhibitor ML385 alone, a GPX4 inhibitor (FIN56 or RSL3) alone, or ML385 combined with a GPX4 inhibitor. We found that combined administration of ML385 and GPX4 inhibitor markedly inhibits the proliferation of AML cell lines compared with either inhibitor alone, and the combination index (CI) indicating that these two inhibitors have synergistic effects (Fig. 3c and d and S4a).

**Fig. 4:**
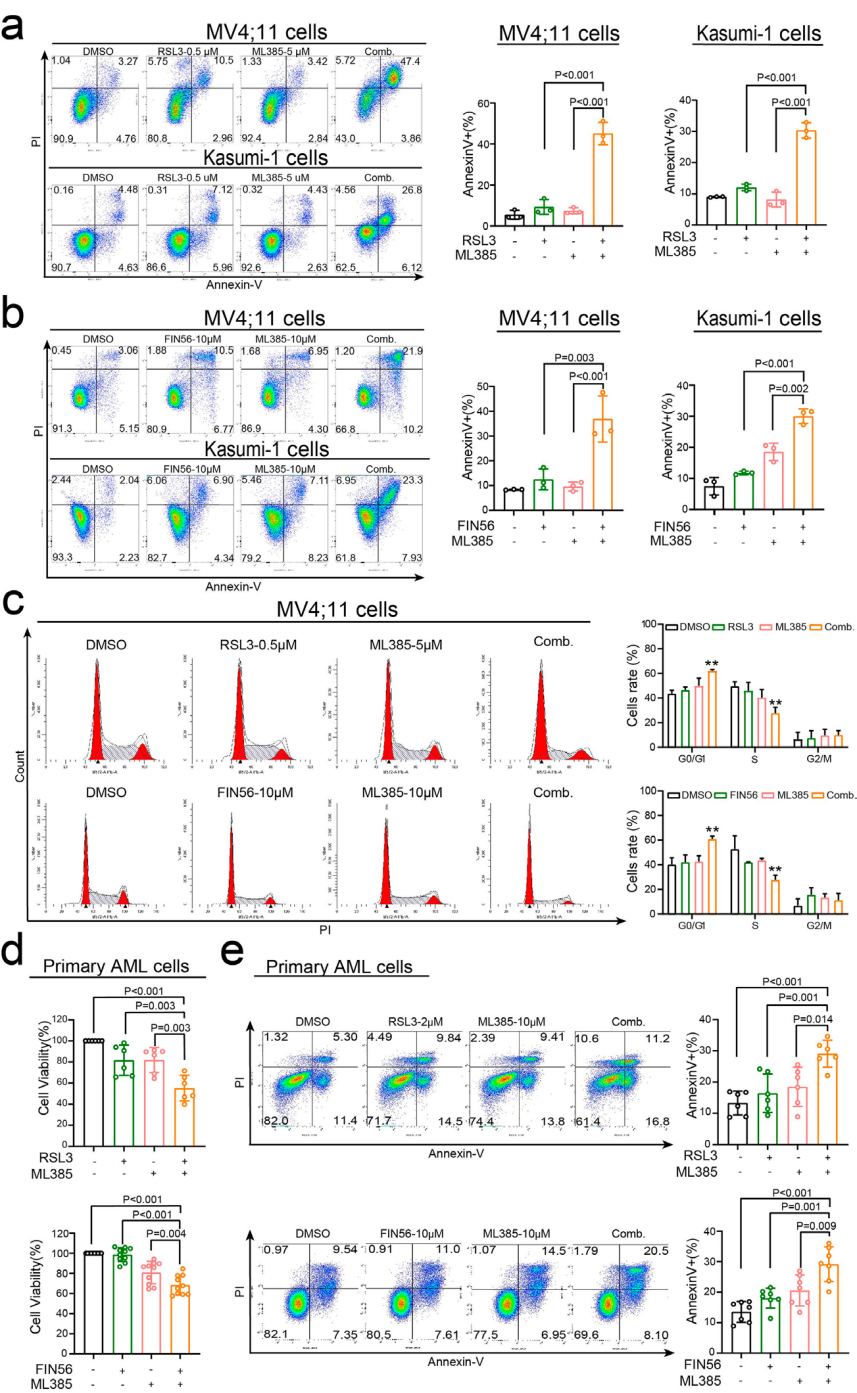
ML385 cooperates with FIN56/RSL3 to induce the cell death of AML cells. (**a**) MV4;11 (upper panel) and Kasumi-1 cells (lower panel) were treated with 0.5 µM RSL3, 5 µM ML385, or the combination of RSL3 and ML385 for 24 h, and the percentage of Annexin V + cells was determined by flow cytometry. (**b**) MV4;11 (upper panel) and Kasumi-1 cells (lower panel) were treated with 10 µM FIN56, 10 µM ML385, or the combination of FIN56 and ML385 for 48 h, and the percentage of Annexin V + cells was determined by flow cytometry. (**c**) MV4;11 cells were co-treated with RSL3 and ML385 (upper panel) or with FIN56 and ML385 (lower panel) at the indicated concentration for 24 h, and the cell cycle was determined by flow cytometry. (**d**) BMMCs from newly diagnosed AML patients were treated with 2 µM RSL3, 10 µM ML385, or a combination of RSL3 and ML385 for 48 h (upper panel), as well as with 10 µM FIN56, 10 µM ML385, or a combination of FIN56 and ML385 for 48 h (lower panel), and cell viability was analyzed using the CCK8 assay. (**e**) BMMCs from newly diagnosed AML patients were treated with 2 µM RSL3, 10 µM ML385, or a combination of RSL3 and ML385 for 48 h (upper panel). In addition, cells were treated with 10 µM FIN56, 10 µM ML385, or a combination of FIN56 and ML385 for 48 h (lower panel). The percentage of Annexin V + cells was then detected using flow cytometry. Data are expressed as the mean ± SD. *n* = 3 or more independent biological replicates, presented as individual points. P value < 0.05 was considered significant (a-e, one-way ANOVA with Bonferroni post hoc test).

Further studies revealed that treatment with ML385 and a GPX4 inhibitor significantly increases the cell death of MV4;11 and Kasumi-1 cells (Fig. 4a, b). In line with these findings, it was observed that ML385 acted in collaboration with Erastin, another ferroptosis inducer, to induce cell death in MV4;11 cells (Figure S4b). Furthermore, our study has uncovered that RSL3 enhances the effectiveness of the primary drugs used to treat AML like Ara-C (Figure S4c). Additionally, we have observed that treatment with a combination of ML385 and GPX4 inhibitors results in the arrest of the cell cycle of MV4;11 cells in the G0/G1 phase, as demonstrated in Fig. 4c. We further examined if the co-administration of ML385 and a GPX4 inhibitor would augment the cytotoxic effects in cells obtained from newly diagnosed AML patients. Comparable to the findings in AML cell lines, the combined use of ML385 and FIN56/RSL3 inhibited the viability and stimulated the cell death of primary AML cells, as depicted in Fig. 4d and e. In summary, these outcomes imply that the combination of inhibiting NRF2 and GPX4 produces a more robust cytotoxic effect on AML cells.

### ML385 cooperates with FIN56/RSL3 to promote ferroptosis of AML cells

**Fig. 5:**
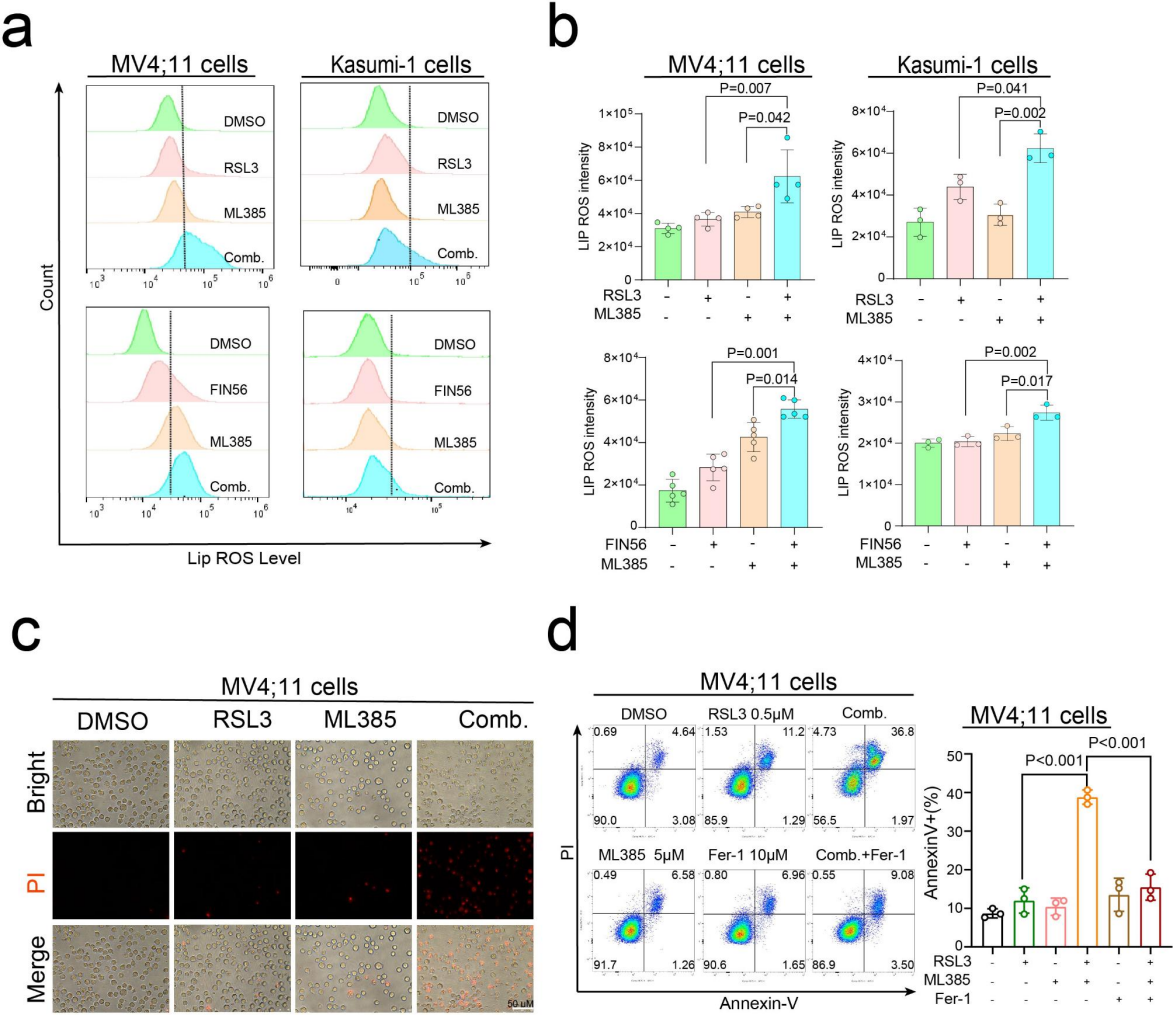
ML385 cooperates with FIN56/RSL3 to promote the ferroptosis of AML cells. (**a**) MV4;11 and Kasumi-1 cells were treated with 5 µM ML385, 0.5 µM RSL3, or a combination of ML385 and RSL3 for 24 h (upper panel). In addition, cells were treated with 10 µM FIN56, 10 µM ML385, or a combination of FIN56 and ML385 for 48 h (lower panel), and the levels of lipid reactive oxygen species (ROS) were determined by flow cytometry using the C11-BODIPY 581/591 probe. (**b**) Histogram showing the levels of lipid ROS. (**c**) MV4;11 cells were treated with 0.5 µM RSL3, 5 µM ML385, or a combination of RSL3 and ML385. To visualize the effect of these compounds on cell death, cells were stained with propidium iodide (PI) and images were obtained using a fluorescence microscope. (**d**) MV4;11 cells were treated with 5 µM ML385, 0.5 µM RSL3, 10 µM ferroptosis inhibitor ferrostatin-1 (Fer-1), or the combination of ML385 and RSL3 with Fer-1 for 24 h, the percentage of Annexin V + cells was determined by flow cytometry. Data are expressed as the mean ± SD. *n* = 3 or more independent biological replicates, presented as individual points. P value < 0.05 was considered significant (b, d, one-way ANOVA with Bonferroni post hoc test).

Previous studies have shown that GPX4 plays a crucial role in ferroptosis and chemoresistance in human cancers. Activation of the NRF2 pathway plays a dominant role in protecting human cancer cells against cell death. To investigate potential synergistic effects of ML385 and GPX4 inhibitors on ferroptosis, we assessed the combined impact of these agents on lipid peroxidation levels using the C11-BODIPY probe. As depicted in Fig. 5a and b, co-treatment with ML385 and GPX4 inhibitors markedly elevated lipid-based ROS accumulation in MV4;11 and Kasumi-1 cells. Consistent with these results, simultaneous treatment with ML385 and RSL3 led to the highest cell death compared to ML385 or RSL3 alone in MV4;11 cells (Fig. 5c). Furthermore, we confirmed that the enhanced cytotoxic effects of ML385 and RSL3 in AML cell lines was blocked by ferrostatin-1 (Fer-1, an inhibitor of ferroptosis) (Fig. 5d). The above data suggested that ML385 may act synergistically with FIN56/RSL3 to induce ferroptosis via the NRF2/GPX4 pathway.

### Synergistic effects of ML385 and FIN56 in downregulating NRF2 and GPX4 expression

**Fig. 6:**
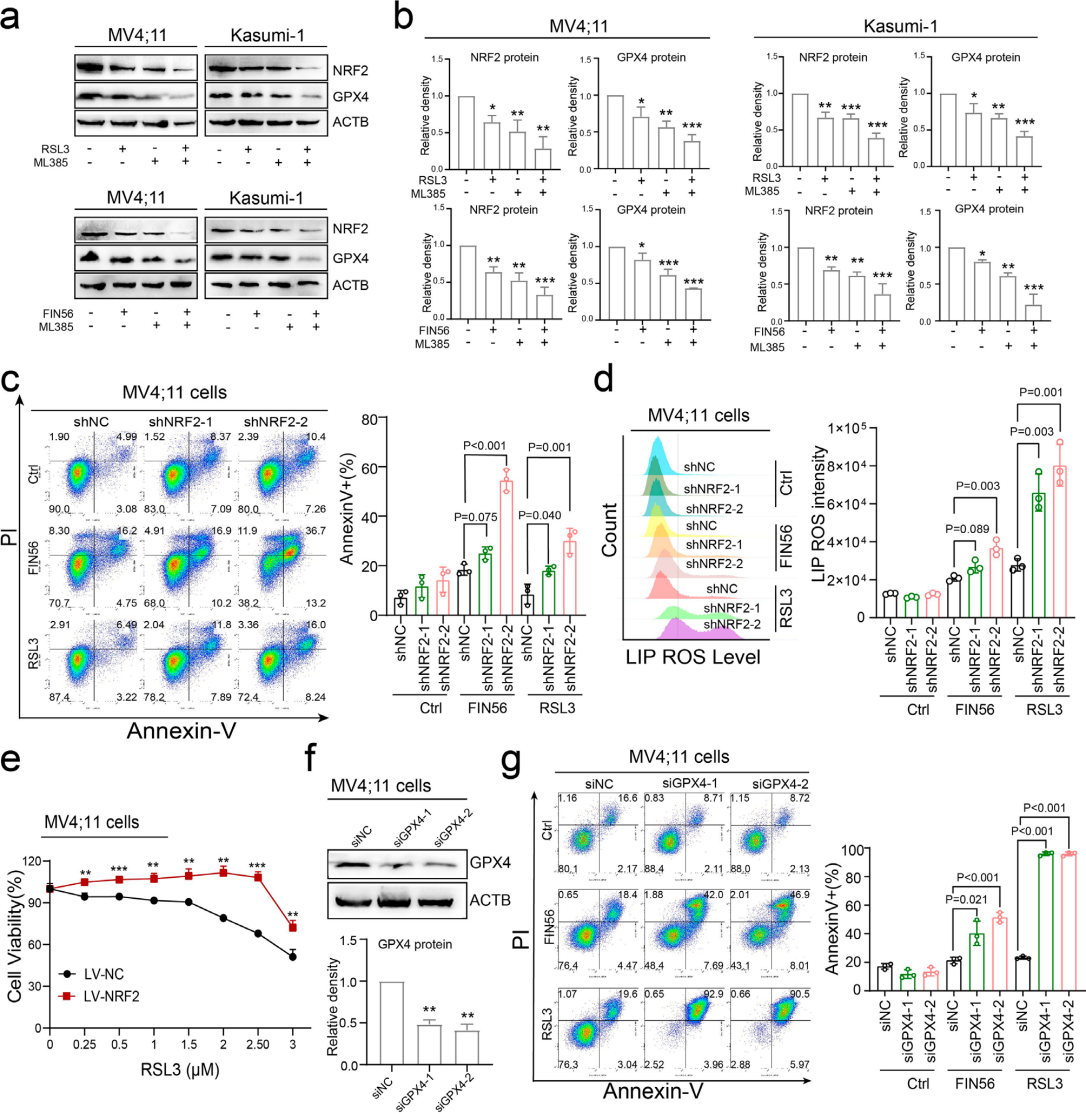
NRF2/GPX4 expression contributes to ferroptosis resistance. (**a**) MV4;11 and Kasumi-1 cells were co-treated with 0.5 µM RSL3 and 5 µM ML385 for 24 h, and the expression levels of NRF2 and GPX4 were detected by western blotting (upper panel). Similarly, MV4;11 and Kasumi-1 cells were co-treated with 10 µM FIN56 and 10 µM ML385 for 48 h, and the expression levels of NRF2 and GPX4 were detected by western blotting (lower panel). (**b**) Western blots were quantified and are presented in the form of a histogram. (**c**) MV4;11 cells transfected with shNRF2 were treated with 10 µM FIN56 or 0.5 µM RSL3 for 48 h, and the percentage of Annexin V + cells was determined by flow cytometry. (**d**) MV4;11 cells transfected with shNRF2 were treated with 10 µM FIN56 or 0.5 µM RSL3 for 24 h, and the lipid ROS levels were determined by flow cytometry using C11-BODIPY 581/591 probe. (**e**) MV4;11 cells transfected with LV-NC or LV-NRF2 vector were treated with the indicated concentrations of RSL3 for 24 h, and the cell viability was analyzed by the CCK8 assay. (**f**) The expression levels of GPX4 and NRF2 in MV4;11 cells transfected with small interfering RNAs (siRNAs) targeting GPX4 (siGPX4) were determined by western blotting. (**g**) MV4;11 cells transfected with siGPX4 were treated with 10 µM FIN56 or 0.5 µM RSL3 for 24 h, and the percentage of Annexin V + cells was analyzed by flow cytometry. Data are expressed as the mean ± SD. *n* = 3 or more independent biological replicates, presented as individual points. P value < 0.05 was considered significant (b-d, f-g, one-way ANOVA with Dunnett’s post hoc test; e, two-tailed unpaired Student’s t test).

To further investigate the synergistic effects of NRF2 and GPX4 inhibition, MV4;11 and Kasumi-1 cells were treated with ML385 or GPX4 inhibitor alone or ML385 combined with GPX4 inhibitor, and the expression of NRF2 and GPX4 was detected by western blotting. As shown in Fig. 6a-b, the GPX4 inhibitor combined with the NRF2 inhibitor resulted in the lowest expression of GPX4 and NRF2. At the same time, transfection of shNRF2 sharply increased the sensitivity of MV4;11 cells to GPX4 inhibitors (Fig. 6c). We next measured the level of intracellular lipid ROS in shNRF2-transfected MV4;11 cells to confirm whether knockdown of NRF2 could increase the oxidation of lipids. As expected, down-regulation of NRF2 promoted GPX4 inhibitors-induced lipid oxidation (Fig. 6d). To investigate the impact of NRF2 expression on AML cells treated with GPX4 inhibitors, we overexpressed NRF2 in MV4;11 cells and treated them with RSL3 at the indicated dose. Our results showed that NRF2 overexpression partially restored the cytotoxic effects of RSL3 on MV4;11 cells, as depicted in Fig. 6e. Additionally, we employed two small interfering RNAs (siRNAs) to target GPX4 and explored its effects on the reactivity of AML cells to ferroptosis (Fig. 6f). Our findings indicated that transfection of siRNA targeting GPX4 significantly increased the sensitivity of MV4;11 cells to RSL3 and FIN56, as demonstrated in Fig. 6g. Overall, our results suggest that targeting NRF2 and GPX4 may enhance the sensitivity of AML cells to ferroptosis.

## Discussion

In this study, we made three significant observations. First, the NRF2 protein induced high constitutive GPX4 levels in AML. Second, GPX4 is one of the most important NRF2 target genes in AML. Third, NRF2/GPX4 attenuates ferroptosis in AML.

Previously, we established that NRF2 plays a vital role in the detoxification of ROS and the resistance to chemotherapy in AML [31]. As such, the primary aim of this study was to investigate the detailed mechanisms of NRF2 in chemoresistance. The NRF2 signaling pathway is notably activated in AML [13, 32], and our prior studies have revealed that the transcriptional activation of the NRF2 signaling pathway leads to high expression of miR-125b in AML [31]. Recent research has also suggested that NRF2 transcription promotes GPX4 expression. In agreement with these findings, we have demonstrated that NRF2 can promote an increase in GPX4 mRNA and protein expression. Our data indicate that GPX4 is one of the most important target genes of NRF2 in AML.

Elevated GPX4 levels are frequently observed in many types of cancer, implying that it may be protumorigenic. Our study has found that GPX4 is highly expressed in AML cells, and elevated GPX4 levels correlate with poor prognosis in AML patients. This suggests that GPX4 may play a critical role in the development and treatment of AML. GPX4 functions as a central defensive enzyme against ferroptosis, and previous studies have indicated that NRF2 may regulate GPX4 expression and be related to ferroptosis in solid cancers [12, 33]. Similarly, we found that GPX4 is directly regulated by NRF2 and that it is resistant to ferroptosis. Combined inhibition of NRF2 with ML385 and GPX4 with RSL3/FIN56 synergistically targets AML cells. Consistent with these findings, ML385 cooperated with another ferroptosis inducer, Erastin, to induce cell death in MV4;11 cells. NRF2 knockdown increased the sensitivity of AML cells to FIN56 and RSL3. At the molecular level, upregulation of GPX4 by NRF2 was found to reduce the cytotoxic effects of these ferroptosis inducers. Still, this resistance could be reversed by adding an NRF2 inhibitor in combination. Similarly, we found significant concurrent down-regulation of NRF2 and GPX4 in response to ML385 + FIN56/RSL3 treatment. Thus, our data suggest that dysregulation of NRF2/GPX4 signaling may be a potential barrier to ferroptosis inducers.

Chemotherapy-induced cell death is often associated with the generation of ROS [34–36]. High levels of ROS can cause oxidative stress and lead to the death of cancer cells [37–40]. However, in AML, chemotherapy resistance is associated with the activation of NRF2. Our previous study showed that chemotherapy increased NRF2 levels in AML cells, and NRF2 activation contributes to chemotherapy resistance [31]. Recently, it has been suggested that ferroptosis is closely linked to the sensitivity of cancer cells to chemotherapy, and induction of ferroptosis may overcome chemotherapy resistance [41]. In fact, several studies have demonstrated that the ferroptosis inducer Erastin can enhance the anticancer activity of Ara-C and doxorubicin [42]. In this study, we also found that RSL3, a GPX4 inhibitor and ferroptosis inducer, enhanced the anticancer activity of Ara-C in AML cells, leading to increased cell death. Our findings suggest that targeting the NRF2/GPX4 pathway and inducing ferroptosis may be an effective strategy for enhancing chemo-sensitivity in AML patients, and may lead to the development of novel combination therapies.

## Conclusions

Our investigation has revealed that the expression of GPX4 in AML is likely attributed to the activity of NRF2 in AML cells. Furthermore, we have demonstrated that the NRF2/GPX4 axis plays a crucial part in ferroptosis and the antileukemia response. Based on these findings, our study provides supporting evidence and a basis for the targeting of NRF2/GPX4 as a strategy for developing therapeutics to improve chemo-sensitivity in AML.

## Electronic supplementary material

Below is the link to the electronic supplementary material.

Supplementary Material 1. Clinical information and primer sequences.

Supplementary Material 2. The KEGG enrichment of 349 NRF2-Chip intersection genes.

Supplementary Material 3. The information of 70 candidate genes.

Supplementary Material 4. The top 20 KEGG enrichments of the 70 candidate genes.

Supplementary Material 5. Supplementary figures.

## Data Availability

The data that support the findings of this study are available from the corresponding author upon reasonable request.
