# Supplementary material for: Targeting NRF2 uncovered an intrinsic susceptibility of acute myeloid leukemia cells to ferroptosis
Source: Exp Hematol Oncol. 2023 May 17;12:47. doi: 10.1186/s40164-023-00411-4 (PMC10189915; doi:10.1186/s40164-023-00411-4)
Supplement: Supplementary file 1 — Supplementary Material 1. Clinical information and primer sequences. [file 40164_2023_411_MOESM1_ESM.docx]

**Supplemental information**

**Supplemental Table 1:** Clinical information.

| **ID** | **Gender** | **Age** | **Used in Figure** | **Subtype** | **Gene type** |
| --- | --- | --- | --- | --- | --- |
| HI-1 | F | 45 | 2c | / | / |
| HI-2 | M | 27 | 2c | / | / |
| HI-3 | F | 19 | 2c | / | / |
| HI-4 | F | 50 | 2c | / | / |
| HI-5 | M | 52 | 2c | / | / |
| HI-6 | M | 38 | 3b | / | / |
| HI-7 | F | 32 | 3b | / | / |
| HI-8 | M | 30 | 3b | / | / |
| AML-1 | F | 51 | 2c | M4 | MLL/ELL |
| AML-2 | F | 32 | 2c | M5 | FLT3-ITD, NPM1 |
| AML-3 | F | 70 | 2c | M1 | ND |
| AML-4 | M | 56 | 2c | / | ND |
| AML-5 | M | 35 | 2c | / | ND |
| AML-6 | F | 62 | 2c, 4d, 4e | M5 | ND |
| AML-7 | M | 68 | 2c, 4d | M5 | ND |
| AML-8 | F | 46 | 2c, 4d, 4e | / | AML1-ETO, KRAS |
| AML-9 | M | 44 | 4d, 4e | M4 | FLT3-TKD, NPM1, NRAS |
| AML-10 | M | 73 | 2c, 4d, 4e | / | ND |
| AML-11 | F | 62 | 2c, 4d | / | / |
| AML-12 | M | 83 | 2c,4d,4e | / | ND |
| AML-13 | F | 62 | 2c, 4d, 4e | M2a | AML1-ETO |
| AML-14 | M | 43 | 2c, 4d, 4e | / | / |
| AML-15 | F | 64 | 2c, 4d, 4e | M2 | ND |
| AML-16 | F | 57 | 2c, 4e, 4e | / | FLT3-TKD, NPM1 |

**Notes: ND: not detected, /: unknown.**

**Supplemental Table 2:** Primer sequences for siRNA and shRNA.

| shRNA | Sense (5' - 3') |
| --- | --- |
| control | Plko.1 |
| shNRF2-1 | CCGGAAGAGTATGAGCTGGAAAAACCTCGAGGTTTTTCCAGCTCATACTCTTTTTTTG |
| shNRF2-2 | CCGGGCTCCTACTGTGATGTGAAATCCTCGAGGATTTCACATCACAGTAGGAGCTTTTTG |
| siNC | siN2142488 |
| siGPX4-1 | UUGUCGAUGAGGAACUUGG |
| siGPX4-2 | GAGGCAAGACCGAAGUAAA |

**Supplemental Table 3:** Primer sequences for qRT-PCR.

| Gene target | Sense (5' - 3') | Antisense (5' - 3') |
| --- | --- | --- |
| ACTB | TTGTTACAGGAAGTCCCTTGCC | ATGCTATCACCTCCCCTGTGTG |
| NRF2 | GAGAGCCCAGTCTTCATTGC | TTGGCTTCTGGACTTGGAAC |
| GPX4 | GAGGCAAGACCGAAGTAAACTAC | CCGAACTGGTTACACGGGAA |
| SLC7A11 | TCTCCAAAGGAGGTTACCTGC | AGACTCCCCTCAGTAAAGTGAC |
| HMOX1 | AAGACTGCGTTCCTGCTCAAC | AAAGCCCTACAGCAACTGTCG |
| GCLC | ATGGAGGTGCAATTAACAGAC | ACTGCATTGCCACCTTTGCA |
| GCLM | TGTCTTGGAATGCACTGTATCTC | CCCAGTAAGGCTGTAAATGCTC |
| FTL | CAGCCTGGTCAATTTGTACCT | GCCAATTCGCGGAAGAAGTG |
| FTHI | CGAGGTGGCCGAATCTTCC | GTTTGTGCAGTTCCAGTAGTGA |
