# Supplementary material for: Targeting NRF2 uncovered an intrinsic susceptibility of acute myeloid leukemia cells to ferroptosis
Source: Exp Hematol Oncol. 2023 May 17;12:47. doi: 10.1186/s40164-023-00411-4 (PMC10189915; doi:10.1186/s40164-023-00411-4)
Supplement: Supplementary file 5 — Supplementary Material 5. Supplementary figures. [file 40164_2023_411_MOESM5_ESM.docx]

**Supplemental Figure**

**
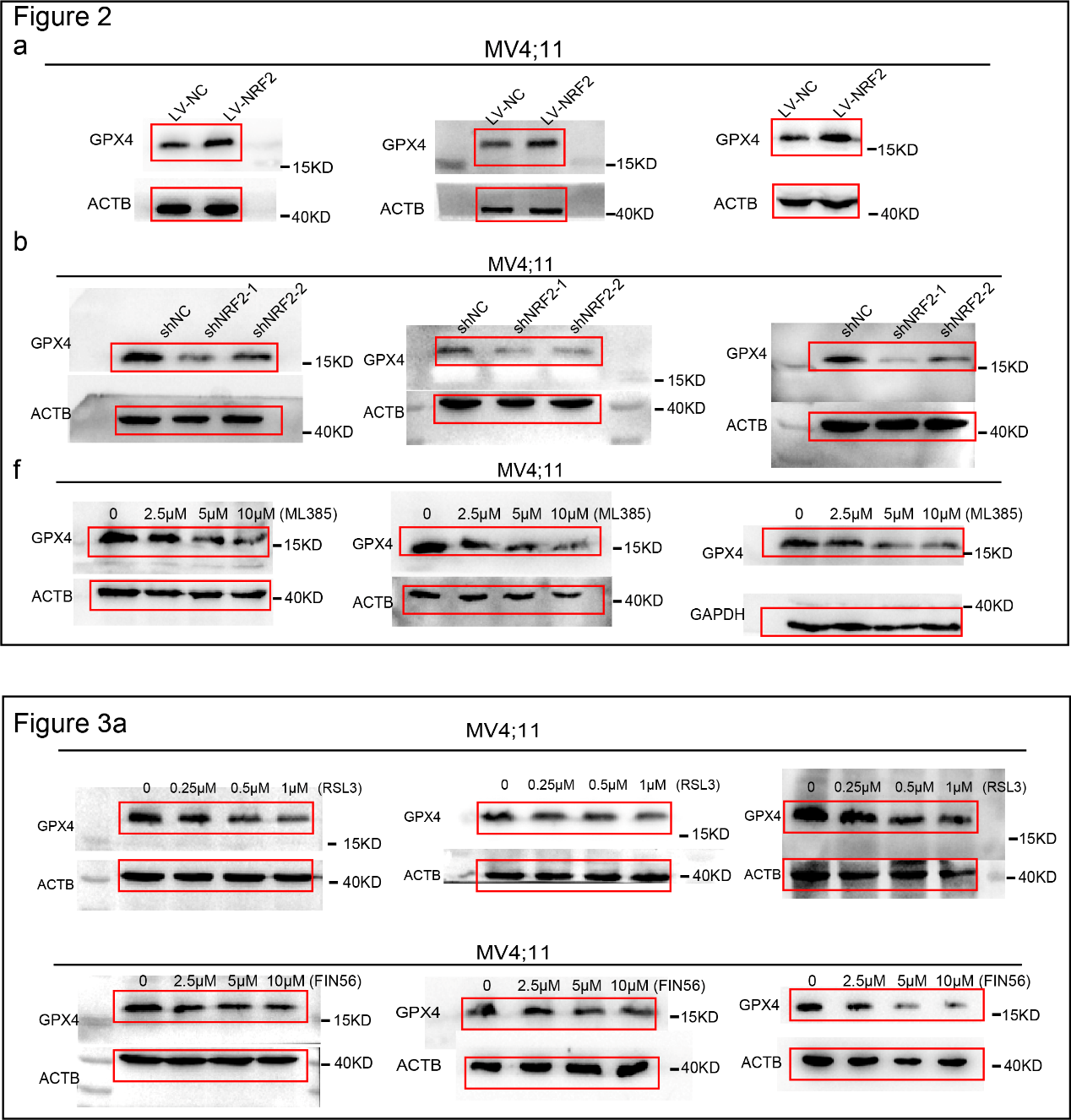

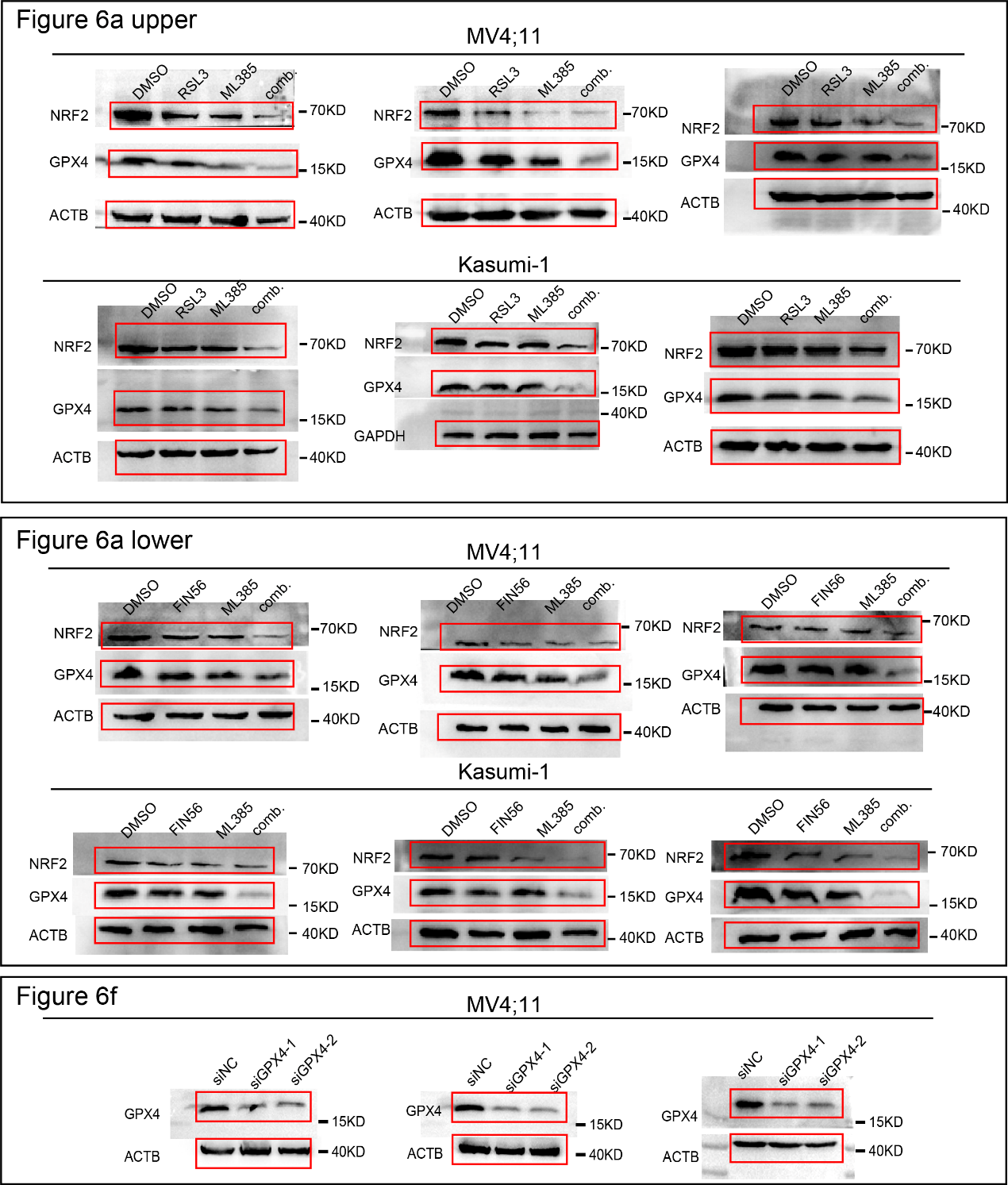
**

**Supplemental Figure 1.** Full length Western Blot in Figure 2-6.


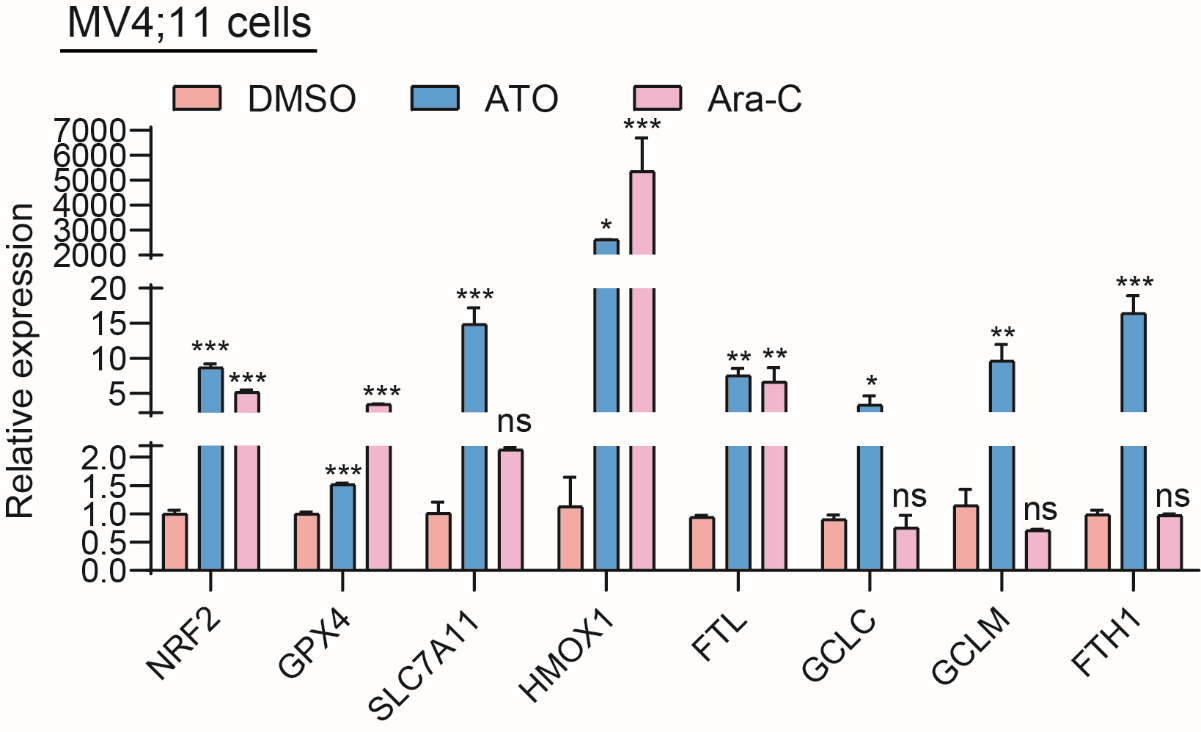


**Supplemental Figure 2.** Effect of ATO and Ara-C on the expression of ferroptosis genes. The expression of genes related to ferroptosis in MV411 cells treated with 1 μM ATO or 1 μM Ara-C for 24 h was detected by qRT-PCR. Data are expressed as the mean ± SD. *n* = 3 or more independent biological replicates, presented as individual points. P value < 0.05 was considered significant (one-way ANOVA with Dunnett's post hoc test).


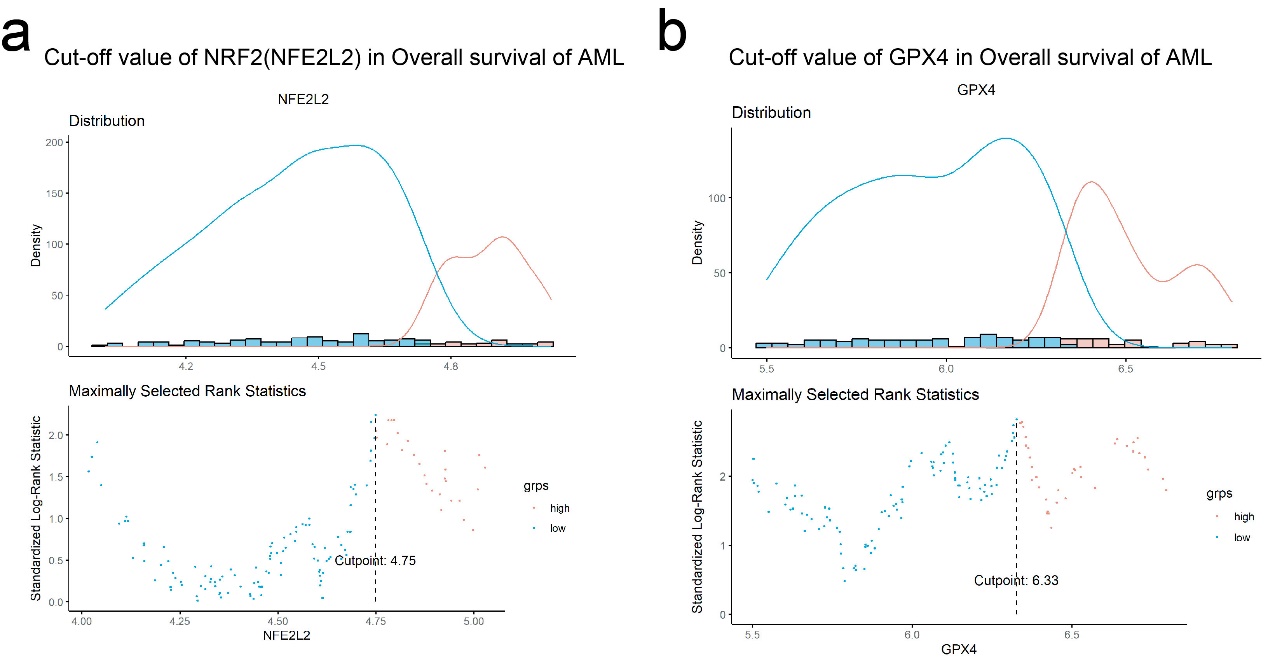


**Supplemental Figure 3.** Cut-off value of NFE2L2 (a) and GPX4 (b) for overall survival analysis in Figure 2e.


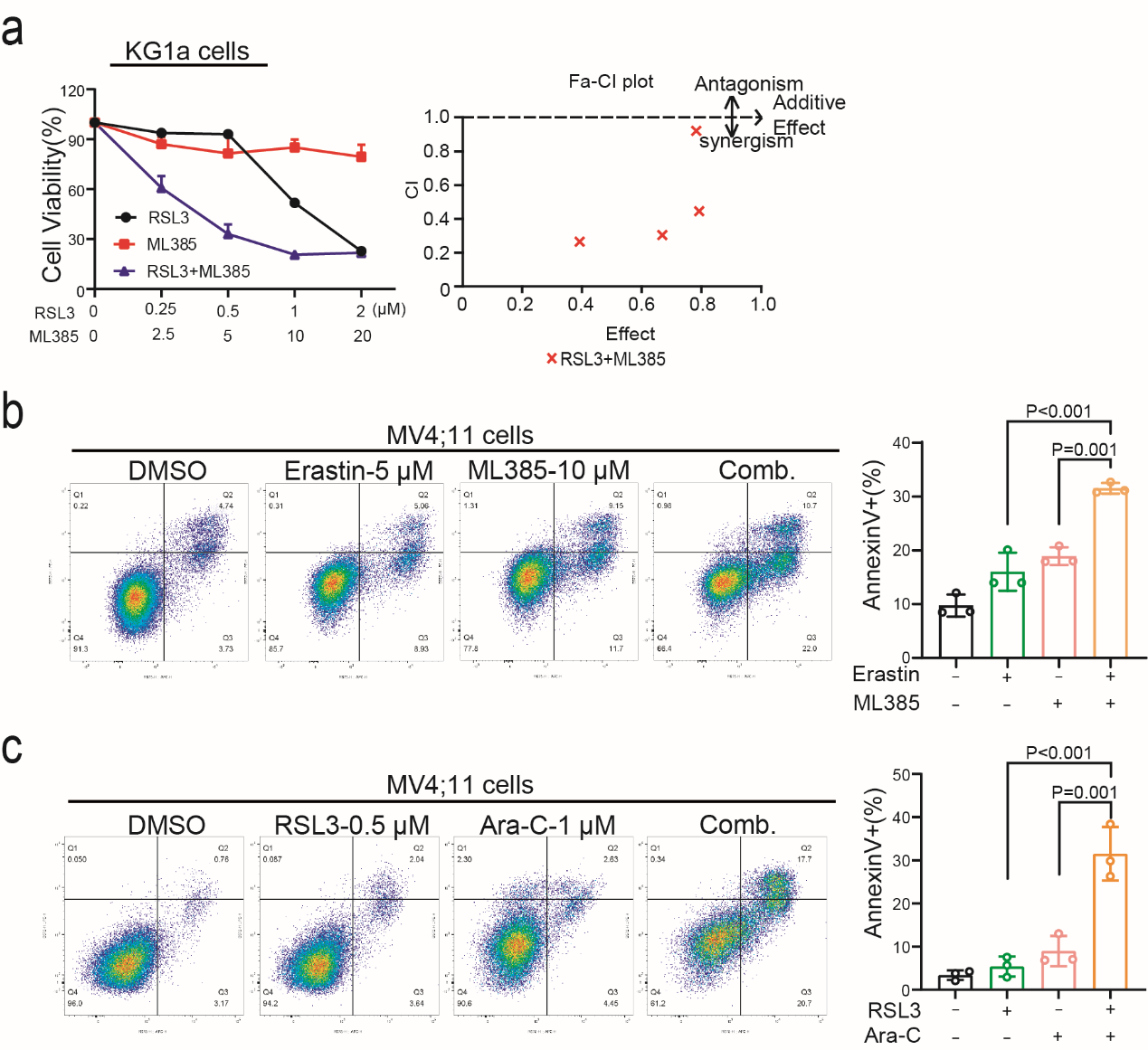


**Supplemental Figure 4.** The potential of ferroptosis inducers in targeting AML cells was evaluated in combination with NRF2 inhibition or chemotherapy. (a) KG1α cells were exposed to ML385 alone, RSL3 alone, or the combination of ML385 and RSL3 at specified concentrations for 24 h, and cell viability was measured by CCK8 assay. Combination index (CI) values were calculated by the Chou-Talalay method. CI value of 1 is indicated by the dashed line, while CI < 1 indicates a synergistic interaction between the two agents in the combination. (b) MV411 cells were co-treated with 5 μM Erastin, 10 μM ML385, or the combination of Erastin and ML385 for 72 hours, and the percentage of Annexin V+ cells was determined by flow cytometry. (c) MV411 cells were treated with 0.5 μM RSL3, 1 μM Ara-C, or the combination of RSL3 and Ara-C for 24 h, and the percentage of Annexin V+ cells was determined by flow cytometry. Data are presented as mean ± SD with n = 3 or more independent biological replicates as individual points. P value < 0.05 was considered significant (b-c one-way ANOVA with Bonferroni post hoc test).
